# Helicobacter pylori cagA, vacA and babA2 genotypes and gastroduodenal diseases: a cross-sectional study from the Mekong Delta of Vietnam

**DOI:** 10.1099/jmm.0.002096

**Published:** 2025-11-18

**Authors:** Thi Hong Nhung Thai, Thai Hoa Nguyen, Thi Mai Ngan Nguyen, Thi Minh Thi Ha

**Affiliations:** 1 Department of Internal Medicine, Faculty of Medicine, Can Tho University of Medicine and Pharmacy, Can Tho, Vietnam; 2 Department of Medical Genetics, Hue University of Medicine and Pharmacy, Hue, Vietnam; 3 Institute of Biomedicine, Hue University of Medicine and Pharmacy, Hue, Vietnam

**Keywords:** *babA2*, *cagA*, gastric precancerous lesions, gastroduodenal disease, *Helicobacter pylori*, *vacA*

## Abstract

**Introduction.** The *cagA* and *vacA* genes encode the CagA and VacA proteins, which are the two main toxins of *Helicobacter pylori*. Regardless of whether the illness is benign or malignant, the majority of Asian *H. pylori* strains are *cagA* (*+*) and *vacA* s1 (*vacA* signal region 1 allele); hence, these genotypes cannot account for the severity of gastroduodenal disease.

**Gap statement.** The *babA2* gene encodes the important adhesin BabA of *H. pylori*, which is crucial for persistent colonization and facilitates the translocation of CagA into host gastric epithelial cells. The synergic interaction of toxins, including CagA, VacA and BabA, could significantly contribute to the pathogenesis of *H. pylori*. The investigation of *cagA*, *vacA* and *babA2* genes in clinical *H. pylori* isolates in Asian nations, particularly Vietnam, is insufficient.

**Aim.** To investigate the *cagA*, *vacA* and *babA2* genotypes to further understand their synergistic interaction in the development of gastroduodenal disease in Vietnamese populations.

**Methodology.** A cross-sectional study was conducted on 169 *H*. *pylori* strains isolated from patients with gastroduodenal disease. The PCR assays were performed to determine the *cagA*, *vacA* and *babA2* genotypes on DNA extracted from cultured *H. pylori* isolates.

**Results.** The research showed that the percentage of the *cagA*(+), *babA2*(+), *vacA* s1m1 and *vacA* s1m2 was 87.6%, 73.4%, 52.1% and 44.4%, respectively. The frequencies of *cagA*(+)/*babA2*(+)/*vacA*s1m1 and *cagA*(+)/*babA2*(+)/*vacA*s1m2 combinations were 44.4% and 28.4 %, respectively. The *cagA*(+)/*babA2*(+)/*vacA*s1m2 combination was associated with peptic ulcer disease [adjusted odds ratio (aOR)=5.53, 95 % confidence interval (CI) 1.09–28.16, *P*=0.039] in male patients and chronic gastritis with precancerous lesions (aOR=5.31, 95 % CI 1.23–22.89, *P*=0.025) in female patients.

**Conclusion.** The *cagA*(+)/*babA2*(+)/*vacA*s1m1 and *cagA*(+)/*babA2*(+)/*vacA*s1m2 combinations were found to be quite prevalent among Vietnamese *H. pylori* strains. The synergistic effect of *cagA*(+), *babA2*(+) and *vacA* s1m2 in increasing the odds of both peptic ulcer disease and gastric precancerous lesions has been observed.

## Introduction

*Helicobacter pylori *is a Gram-negative bacterium and infects more than 50% of the worldwide population [1]. It is the leading cause of chronic gastritis, peptic ulcers and gastric cancer [2]. According to Correa’s cascade of gastric carcinogenesis, this bacterium causes chronic gastritis, followed by gastric precancerous lesion stages, including atrophy, intestinal metaplasia, dysplasia and, finally, gastric cancer [3,4]. Although the rate of the population infected with *H. pylori* is high, in fact, only a small number of them progress to peptic ulcers (10–20 %) or gastric cancer (1–2 %) [5,7]. Since *H. pylori* virulence is one of the three main factors in the pathogenesis of gastroduodenal diseases, the difference in *H. pylori*-related clinical outcomes may be explained partly by the distinction of molecular characteristics of *H. pylori* strains. This pathogen had several virulence factors for successful colonization and chronic infection [8].

The *cagA* gene encodes the CagA protein, a well-known toxin of *H. pylori*. CagA is translocated into gastric epithelial cells, undergoes phosphorylation on tyrosine by Scr and Ab1 kinases and then activates SHP2 tyrosine phosphatase, which leads to impairing several signalling pathways, causing morphological cellular aberrations [9,10]. CagA has been considered the key virulence factor affecting *H. pylori*-related clinical outcomes, especially peptic ulcers or gastric cancer. This conclusion may not be correct for *H. pylori* strains originating from East Asian countries. Most East Asian *H. pylori* strains possess *cagA*(+) genes (about >90 %) but are not equivalent to the rate of severe clinical outcomes [11]. The combined role of other virulence genes of *H. pylori* with *cagA* in developing gastroduodenal diseases has been questioned.

The *vacA* gene encodes the VacA protein, which was usually studied with the *cagA* gene associated with gastroduodenal diseases [12,13]. VacA can induce vacuole formation in the gastric epithelial cells, apoptosis stimulation and T‐cell proliferation block [14]. *H. pylori* strains carry the *vacA* genes with the genetic variations in the signal (s) region with two alleles (s1 and s2) and the middle (m) region with two alleles (m1 and m2), and the combination of variations in the two regions of s and m of the *vacA* gene leads to different vacuolating abilities in *H. pylori* strains [14]. The s1m1 subtype in particular exhibits high vacuolating activity, the s1m2 subtype has intermediate vacuolating activity and the s2m2 subtype has no vacuolating activity [15].

Among several virulence genes of *H. pylori*, in addition to the two well-known genes *cagA* and *vacA*, genes encoding outer membrane proteins that function as adhesins have also been reported [16]. The *babA2* gene encodes the BabA protein, which is one of the most important adhesins of *H. pylori* [17]. The BabA binds with Leb (ABO/Leb blood group antigens) on the surface of gastric epithelial cells and then enhances the translocation of CagA into host gastric cells [10,18]. Regardless of whether the illness is benign or malignant, the majority of East Asian *H. pylori* strains are *cagA* (*+*) and *vacA* s1 [11]; hence, these genotypes cannot account for the severity of gastroduodenal disease. This study aimed to investigate the *cagA*, *vacA* and *babA2* genotypes to further understand their synergistic interaction in the development of gastroduodenal disease in Vietnamese populations.

## Methods

### Study design and participants

The cross-sectional study was carried out at the Can Tho University of Medicine and Pharmacy Hospital, which serves many patients from many provinces in the Mekong Delta region of Vietnam.

**Fig. 1.**
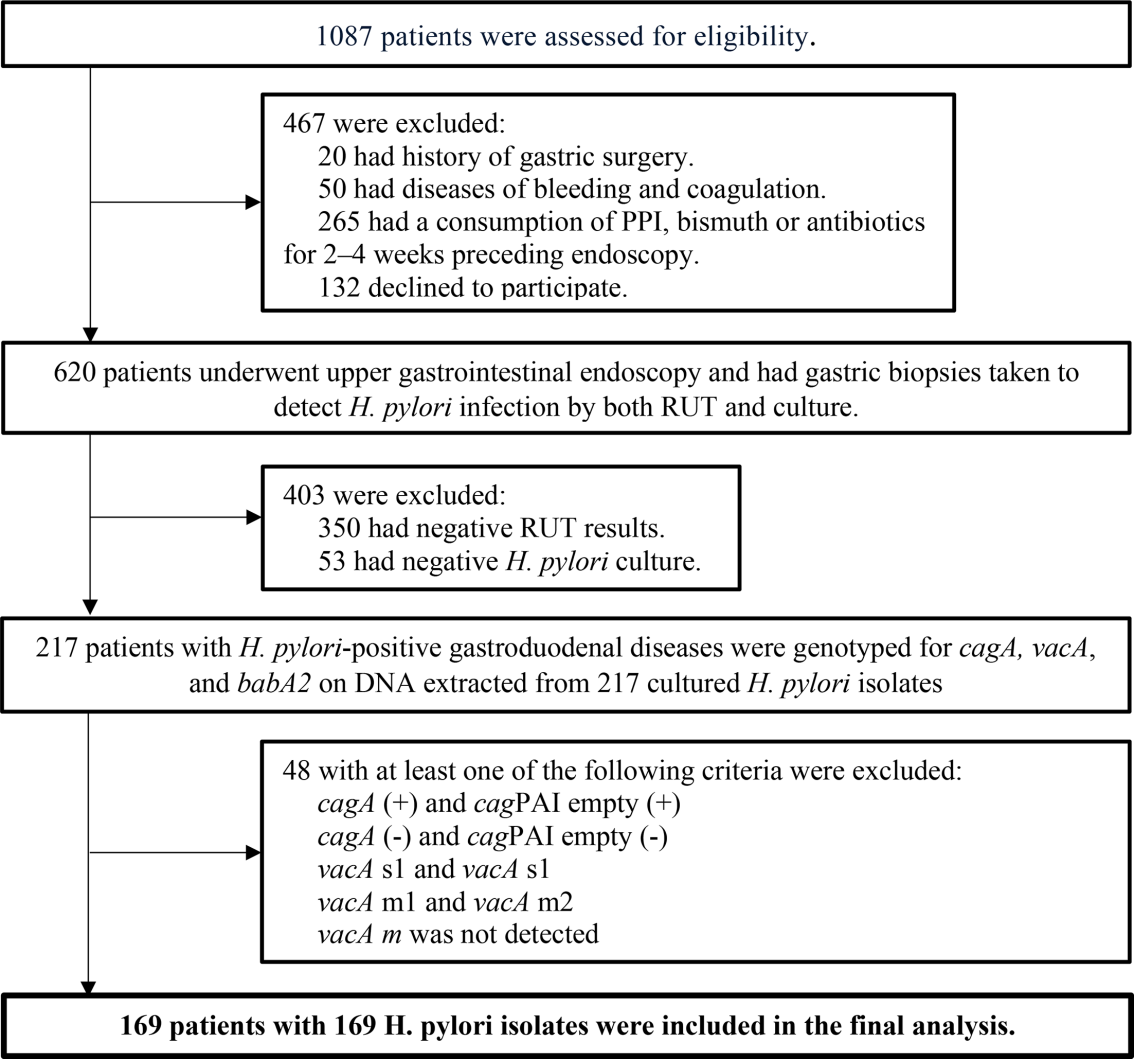
Flowchart of recruitment.

All patients with dyspepsia who were indicated for upper gastrointestinal endoscopy were assessed for eligibility. The inclusion criteria included patients who were diagnosed with chronic gastritis or peptic ulcer disease with positive *H. pylori* infection in both the rapid urease test and culture. The exclusion criteria included patients with a history of upper gastrointestinal surgery, disorders of coagulation, consumption of antibiotics/bismuth within 4 weeks or proton pump inhibitors within 2 weeks prior to the endoscopy. We also excluded patients with PCR results indicating mixed *H. pylori* strains, as detailed in Fig. 1. The flowchart of patient selection is described in detail in Fig. 1.

Each studied patient underwent an upper gastrointestinal endoscopy to visualize the endoscopic lesions and take gastric biopsies for the rapid urease test, culture or histopathology according to a protocol described in our previous study [19]. Patients in this study were divided into three groups of gastroduodenal disease based on the endoscopic and histopathological findings, including non-atrophic chronic gastritis, peptic ulcers and chronic gastritis with precancerous lesions (atrophy, intestinal metaplasia or dysplasia). In addition, we categorized the studied patients into two age groups: <40 and ≥40 years, based on previous studies that reported an increased risk of atrophy, intestinal metaplasia and peptic ulcer in individuals aged 40 years and above [20,21].

### Culture of *H. pylori* and DNA extraction

Two gastric biopsies from the antrum and corpus were placed into 0.5 ml of transportation medium (20% glycerol and 0.9% NaCl in Milli-Q water), kept at 2–8 °C and delivered to the microbiology laboratory within 2–4 h. The biopsy fragments were then ground in a culture medium (100 µl of brain heart infusion solution supplemented with 10% foetal bovine serum), and the homogenate was spread onto agar plates supplemented with 10% lysed horse blood and an antibiotic mixture, including vancomycin (10 mg l^−1^), trimethoprim (5 mg l^−1^), amphotericin B (5 mg l^−1^) and polymyxin B (2,500 IU/L). These plates were incubated in a microaerobic atmosphere consisting of 85% N₂, 10% CO₂ and 5% O₂ at 37 °C for 3–10 days. *H. pylori* colonies were confirmed based on the morphology, Gram-negative curved rod-like, seabird-like or spiral bacterium and the positivity for oxidase, catalase and urease. *H. pylori* colonies were stored at −20 °C in TE buffer solution until DNA extraction, which was performed as previously described [22].

### Genotyping of *H. pylori cagA*, *vacA* and *babA2* genes

The *cagA* status and *vacA* sm genotypes were determined by the PCR method previously described [23]. Two PCR assays were conducted to identify the cagA gene status, including the first primer pairs, cag2: GGAACCCTAGTCGGTAATG and CAGTR: GCTTTAGCTTCTGAYACYGC, which yielded products ranging in size from 450 to 550 bp [24,25], and the second primer pairs, cag5c-F: 5′-GTTGATAACGCTGTCGCTTC-3′ and cag3c-R: 5′-GGGTTGTATGATATTTTCCATAA-3′, which yielded a 350-bp product [26]. Additionally, a PCR assay using the primers Luni1: ACATTTTGGCTAAATAAACGCTG and R5280: GGTTGCACGCATTTTCCCTTAATC was performed for all samples to determine the ‘cagPAI empty site’, which yielded a 550-bp product [27]. The PCR assays of the *cagA* gene or the ‘*cag*PAI empty site’ were performed in a total volume of 25 µl containing 12.5 µl of OneTaq 2×Master Mix (New England BioLabs, UK), 1 µl of each forward and reverse primer (10 pmol µl^−1^), 100 ng DNA template and nuclease-free water with a condition of 95 °C 5 min for initial denaturation, followed by 30 cycles including 94 °C 1 min, 53 °C 1 min and 72 °C 1 min and final extension at 72 °C 10 min.

A multiplex PCR assay was performed for genotyping *vacA* sm using VA1-F: 5′-ATGGAAATACAACAAACACAC-3′ and VA1-R: 5′-CTGCTTGAATGCGCCAAAC-3′ primers specific for the s1 allele of 259 bp and s2 allele of 286 bp and VAG-F: 5′-CAATCTGTCCAATCAAGCGAG-3′ and VAG-R: 5′-GCGTCAAAATAATTCCAAGG-3′ primers specific for the m1 allele of 567 bp and m2 allele of 642 bp [23]. If it failed with multiplex PCR, a simplex PCR assay was conducted using the same specific primers. The PCR reaction was done in a total volume of 25 µl containing 12.5 µl of OneTaq 2×Master Mix (New England BioLabs, UK), 1 µl of each forward and reverse primer (10 pmol/ µL), 100 ng DNA template and nuclease-free water with condition of 95 °C 5 min; 94 °C 1 min, 52 °C 1 min, 72 °C 1 min; and 72 °C 10 min (30 cycles).

The *babA2* status was identified by PCR with the primers of *babA2*-F: AATCCAAAAAGGAGAAAAAGTATGAAA and *babA2*-R: TGTTAGTGATTTCGGTGTAGGACA, which yielded an 832-bp product [28]. This PCR reaction was performed in a total volume of 25 µl containing 5 µl 5′ FIREPol^®^ Master Mix (Solis BioDyne, Estonia), 1 µl of each forward and reverse primer (10 pmol µl^−1^), 100 ng DNA template and nuclease-free water with the condition of 95 °C 5 min; 94 °C 30 s, 50 °C 30 s, 72 °C 1 min; and 72 °C 10 min (35 cycles).

**Fig. 2.**
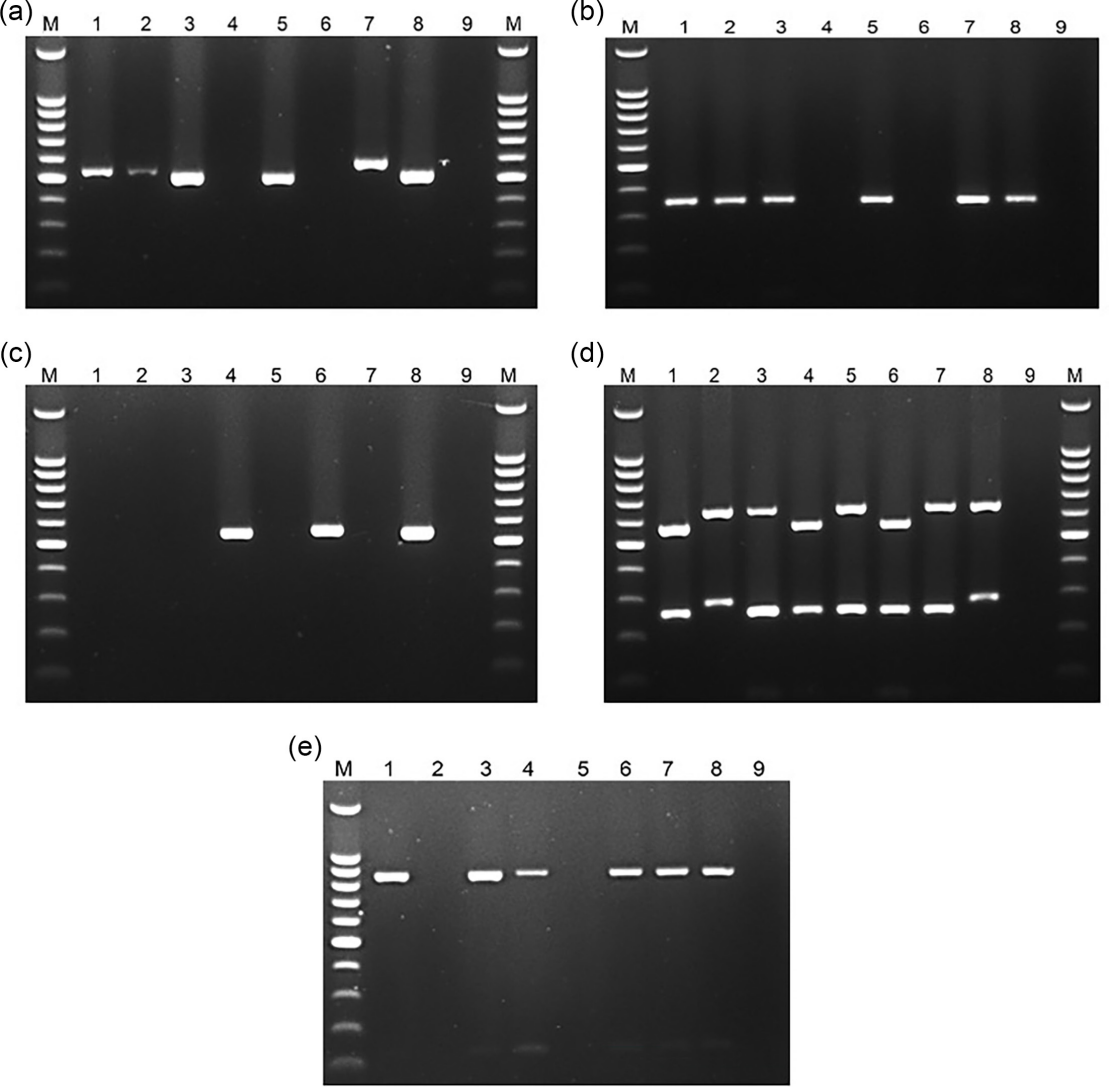
Agarose gel electrophoresis image of the PCR products. Lanes M: 100 bp DNA ladder (Promega Corp., Madison, WI, USA). (a, b and c) Amplification products of the *cagA* gene using primers cag2 and CAGTR, cag5c-F and cag3c-R and amplification products using primers Luni1 and R5280 specific for the ‘cagPAI empty site’, respectively [lanes 1, 2, 3, 5, 7: *cagA*(+) and *cag*PAI empty (−); lanes 4, 6: *cagA* (−) and *cag*PAI empty (+); lane 8: positive control; lane 9: negative control]. (d) Amplification products of *vacA* sm (lanes 1, 4: s1m1; lanes 3, 5: s1m2; lane 2: s2m2; lane 6: *vacA* s1m1 control; lane 7: *vacA* s1m2 control; lane 8: *vacA* s2m2 control; lane 9: negative control). (e): Amplification products of the *babA2* gene [lanes 1, 3, 4, 6, 7: *babA2* (+); lanes 2, 5: *babA2* (−); lane 8: positive control; lane 9: negative control].

Sterile water was used as a negative control, and the DNA samples of *cagA*, *vacA* sm or *babA2*-positive *H. pylori* strain previously identified were used as positive controls. PCR products were examined by electrophoresis at 80 V for 1 h 15 min in 1% agarose with SafeView™ Classic (abm, Canada) added as a DNA staining agent (Fig. 2). The PCR assay was performed in a SureCycler 8800 (Agilent Technologies, Malaysia) at the Department of Medical Genetics, University of Medicine and Pharmacy, Hue University, Vietnam.

### Analysis of PCR results of *cagA*, *vacA* and *babA2* genes

The *cagA*-positive *H. pylori* samples were confirmed when at least one of the two PCR assays (using the first primer pair and the second primer pair, respectively) was positive, and the PCR assay for the cagPAI empty site was negative.

*H. pylori vacA* sm genotypes were confirmed based on the sizes of PCR products (using primer pair VA1-F and VA1-R, VAG-F and VAG-R), including *vacA* s1 or s2 and *vacA* m1 or m2.

The *babA2*(+)-*H. pylori* strain was determined when the PCR assay using primers of *babA2*-R and *babA2*-F was positive.

Samples with at least one of the following characteristics were defined as mixed *H. pylori* strains and were excluded from the study: (i) at least one of the two PCR assays (using primers cag2 and CAGTR, primers cag5c-F and cag3c-R, respectively) was positive, while the PCR assay for the cagPAI empty site was also positive, and vice versa; and/or (ii) the PCR results showed the presence of both vacA s products (s1 and s2) or both vacA m products (m1 and m2).

### Data analysis

The data were processed using SPSS Statistics 26.0. The distribution of a categorical variable in a group was compared with the distribution in another group using the chi-squared test or Fisher’s exact test if the expected values were too low. We performed a multivariable logistic regression after adjusting for age group and gender to investigate the association between each *cagA* gene, *vacA* genotypes and *babA2* gene and gastroduodenal diseases. A *P*-value less than 0.05 was considered statistically significant. Odds ratio and 95% confidence interval (CI) were used to estimate the risk.

## Results

### General characteristics of the study participants

This study included 169 patients with *H. pylori*-positive gastroduodenal diseases (Table 1). Among them, 83/169 (49.1 %) were males, and 86/169 (50.9 %) were females, with 99/169 (58.6 %) of patients aged 40 years and older. Regarding gastroduodenal diseases, 93/169 (55 %) of the patients had chronic gastritis with precancerous lesions.

**Table 1.** The demographic and clinical characteristics of 169* H. pylori*-positive patients with gastroduodenal diseases

Characteristics	Results
**Gender, *n* (%**)	
Male	83 (49.1)
Female	86 (50.9)
**Age group, *n* (%**)	
< 40 years old	70 (41.4)
≥ 40 years old	99 (58.6)
**Gastroduodenal disease, *n* (%**)	
Non-atrophic chronic gastritis	30 (17.8)
Peptic ulcers	46 (27.2)
Chronic gastritis with precancerous lesions	93 (55.0)

### The prevalence of *cagA*, *vacA* and *babA2* genotypes

The prevalence of the *cagA*, *vacA* and *babA2* genotypes among 169 *H*. *pylori* strains is listed in Table 2. The *cagA* gene was detected in 87.6%(148/169) of the strains. The *babA2* gene was present in 124 out of 169 isolates (73.4%).

**Table 2.** The prevalence of *cagA*, *vacA* and *babA2* genes in 169* H. pylori* isolates

Gene	Genotype	*n* (%)	P*
*cagA*	*cagA (+*)	148 (87.6)	<0.001
	*cagA* (−)	21 (12.4)	
*babA2*	*babA2 (+*)	124 (73.4)	<0.001
	*babA2* (−)	45 (26.6)	
*vacA* s	s1	163 (96.4)	<0.001
	s2	6 (3.6)	
*vacA* m	m1	88 (52.1)	0.59
	m2	81 (47.9)	
*vacA* sm	s1m1	88 (52.1)	
	s1m2	75 (44.4)	<0.001
	s2m2	6 (3.6)	
Combination of *cagA/babA2/vacA* sm	*cagA*(+)*/babA2*(+)*/vacA* s1m1	75 (44.4)	
	*cagA*(+)*/babA2*(+)*/vacA* s1m2	48 (28.4)	0.009
	Other combinations	46 (27.2)	

*One-sample chi-square test.

The distribution of *vacA* alleles was as follows: *vacA* s1m1 accounted for 52.1%, *vacA* s1m2 for 44.4% and *vacA* s2m2 for only 3.6%. Most *H. pylori* strains carried the *vacA* s1 allele (96.4%, 163/169), while the *vacA* s2 allele was found in only 3.6%(6/169). However, there was no significant difference between the prevalence of the *vacA* m1 (52.1%, 88/169) and *vacA* m2 (47.9%, 81/169) alleles (*P*=0.590) (Table 2).

Statistically significant associations were observed between *vacA* sm and *babA2* genotypes and *cagA* genotype (Table 3). Notably, the *vacA* s1m1 genotype was exclusively present in *cagA* (+) *H. pylori* strains, and 80% of *vacA* s1m2 strains possessed the *cagA* gene, whereas *vacA* s2m2 genotypes were absent in *cagA* (+) strains (*P*<0.001). Besides, 97.6% of the strains carrying babA2 (+) were cagA (+), whereas only 60% of the babA2 (−) strains were cagA (+) (Table 3). We found that the highly virulent triple-positive gene combinations of *cagA*(+)/*babA2*(+)/*vacA s1m1* and *cagA*(+)/*babA2*(+)/*vacA s1m2* accounted for high proportions, with 44.4% and 28.4 %, respectively (Table 2).

**Table 3.** The association between the *cagA*, *vacA* and *babA2* genotypes

*Genotype*	*cagA* (+), *n* (%)	*cagA* (−)*, n* (%)	*P* **-value**
*vacA*			<0.001*
s1m1	88 (100.0)	0 (0)	
s1m2	60 (80.0)	15 (20.0)	
s2m2	0 (0)	6 (100.0)	
*babA2*			
*babA2* (+)	121 (97.6)	3 (2.4)	<0.001*
*babA2* (−)	27 (60.0)	18 (40.0)	

*Chi-square test.

### Association between *cagA*, *vacA* and *babA2* genotypes of *H. pylori* and gastroduodenal diseases

When analysing the association between each gene of *cagA*, *vacA* and *babA2* and gastroduodenal diseases by multivariable logistic regression adjusted by gender and age group, we found that only the *cagA* (+) genotype was associated with a 13.00-fold increase in the odds of peptic ulcer (95% CI 1.41–120.09, *P*=0.024), while the *vacA s1m1*, *s1m2* genotypes and the *babA2* (+) gene showed no association with clinical outcomes (Table 4). Interestingly, our findings revealed that the *cagA*(+)*/babA2*(+)*/vacA*s1m2 combination was associated with a higher odds of peptic ulcer [adjusted odds ratio (aOR)=5.53, 95% CI1.09–28.16, *P*=0.039] in male patients and chronic gastritis with precancerous lesions (aOR=5.31, 95% CI 1.23–22.89, *P*=0.025) in female patients (Table 5).

**Table 4.** The association between each *H. pylori babA2, cagA* and *vacA* gene and gastroduodenal diseases: the result of multivariable logistic analysis after adjusting for age group and gender

Genotype	NACG	PUD	PCL	PUD vs. NACG		PCL vs. NACG	
	*n*=30	*n*=46	*n*=93	aOR (95% CI)	*P*-value	aOR (95% CI)	*P*-value
*babA2* (+)	19	34	71	1.32 (0.45–3.89)	0.619	1.87 (0.77–4.52)	0.165
*babA2* (−)	11	12	22	1		1	
*cagA* (+)	22	45	81	13.00 (1.41–120.09)	0.024	2.45 (0.89–6.75)	0.082
*cagA* (−)	8	1	12	1		1	
*vacA* s1m1	18	24	46	2.24 (0.15–34.41)	0.563	1.72 (0.26–11.20)	0.571
*vacA* s1m2	10	21	44	3.36 (0.21–54.07)	0.393	3.02 (0.43–20.95)	0.264
*vacA* s2m2	2	1	3	1		1	

NACG, Non-atrophic chronic gastritis; PCL, chronic gastritis with precancerous lesions; PUD, peptic ulcer disease.

**Table 5.** The association between the *cagA*/*vacA*/*babA2* gene combination and gastroduodenal diseases stratified by gender: the result of multivariable logistic analysis after adjusting for age group

Gender	Genotype	NACG	PUD	PCL	PUD vs. NACG		PCL vs. NACG	
					aOR (95 % CI)	***P*-value**	aOR (95 % CI)	***P*-value**
Male	*cagA*(+)*/babA2*(+)*/vacA*s1m1	8	10	20	0.76 (0.21–2.72)	0.669	1.57 (0.52–4.76)	0.422
	*cagA*(+)*/babA2*(+)*/vacA*s1m2 Other combinations	1 4	13 8	10 9	5.53 (1.09–28.16) 1	**0.039**	4.33 (0.89–20.99)	0.069
Female	*cagA*(+)*/babA2*(+)*/vacA*s1m1	8	7	22	0.83 (0.19–3.54)	0.800	1.17 (0.52–3.31)	0.763
	*cagA*(+)*/babA2*(+)*/vacA*s1m2	2	3	19	1.51 (0.20–11.28)	0.690	5.31 (1.23–22.89)	**0.025**
	Other combinations	7	5	13	1			

The p-values in bold indicate statistical significance.

NACG, Non-atrophic chronic gastritis; PCL, chronic gastritis with precancerous lesions; PUD, peptic ulcer disease.

## Discussion

*H. pylori* is the predominant cause of most gastroduodenal diseases, primarily due to the effects of its various virulence factors. Among these factors, the two typical toxin-encoding genes, *cagA* and *vacA*, have been extensively studied and proven to be associated with severe gastroduodenal diseases. However, this association appears inconsistent in East Asia, where most *H. pylori* strains carry the *cagA* gene and *vacA* s1, regardless of gastroduodenal disease severity [11]. This fact has raised the question of whether other virulence factors act synergistically with *cagA* and *vacA* in the development of gastroduodenal diseases in East Asian populations. Our study on *H. pylori cagA*, *vacA* and *babA2* initially demonstrated a strong association between these genes and their synergistic effects on the development of gastroduodenal diseases.

The DNA extraction for investigating virulence genes was performed from cultured *H. pylori* isolates. Despite the fact that the culture technique is complex and costly, we carried it out to reduce the occurrence of mixed *H. pylori* strains, which are common in gastric mucosa biopsies, and to ensure the highest quantity and quality of DNA for PCR testing [29,31]. Besides, we also relied on the results of the PCR assays, which have been described in detail in the ‘Methods’ section, to exclude mixed *H. pylori* samples. Our study found that the rate of *H. pylori cagA* (*+*) was 87.6%. This result is lower than those of several studies, which all reported a *cagA-*positivity prevalence above 90%, including several studies in Hanoi City, Vietnam (96.2%) [20], in Thailand (98.2%) [32] and in China (97%) [33]. This difference may be explained by the geographical variations in *H. pylori cagA-*positivity prevalence and the definition criteria. In detail, our study confirmed that the *cagA* gene was positive when both PCR assays were positive for the *cagA* gene and negative for the *cagPAI* empty site, whereas the above-mentioned studies were defined only based on a positive PCR result for the *cagA* gene [20,32, 33]. This fact has been shown by a study conducted in Hue City, Vietnam, which defined *cagA* (+) strains similarly to our study and recorded the rate of *cagA* (+) as 77.6% [23]. Moreover, although our study used cultured *H. pylori* isolates for DNA extraction, we still identified and excluded 17 out of the initial 217 samples (7.8%) that tested both the *cagA* (*+*) gene and the *cagPAI* empty (+), indicating the presence of mixed *H. pylori* strains, which has also been reported in a previous study [27]. These findings highlight the necessity of simultaneously performing PCR assays targeting both *cagA* and the *cagPAI* empty site to minimize misclassification resulting from mixed *H. pylori* infections.

The *vacA* gene encodes the VacA protein, a vacuolating cytotoxin, which is present in all *H. pylori* strains with different polymorphic genotypes. The vacuolating ability of *vacA* genotypes is determined by the combination of variations in the s and m regions, with s1m1 exhibiting high vacuolating activity, s1m2 showing intermediate activity and s2m2 with no vacuolating activity [6]. Our findings reveal that *vacA* s1 accounted for the majority of studied *H. pylori* strains (96.4%), which is consistent with reports from a Vietnamese study (98.1%) [23], a Peruvian study (94.9%) [34] and a Korean study (100%) [35]. However, regarding *vacA* m, our study identified a high prevalence of *vacA* m2 (47.9%), which is similar to another Vietnamese study (55.1%) [23], but different from the Peruvian study (18.3%) [34] and the Korean study (6%) [35]. This indicates that the geographical diversity of *vacA* m is more pronounced than that of *vacA* s. A previous study has concluded that in East Asia, differences in *H. pylori*-related clinical outcomes could not be explained by the type of s region present, as most *H. pylori* strains carry *vacA* s1 [11]. Our results suggest the hypothesis that *vacA* m may serve as a better indicator genotype for gastroduodenal disease than *vacA* s.

Additionally, our findings indicate that most of the studied *H. pylori* strains carry highly virulent *vacA* sm genotypes, including s1m1 (52.1%) and s1m2 (44.4%), which is consistent with the high virulence of East Asian *H. pylori* strains [36]. Moreover, we observed a significant difference in the distribution of *vacA* sm according to *cagA* genotype, with all *vacA* s1m1 strains present in *cagA* (+) strains (100%), whereas all *vacA* s2m2 strains were found in *cagA*-negative strains (Table 3), similar to a previous study’s conclusions [11].

The *babA2* gene encodes the BabA protein, a crucial outer membrane adhesin that facilitates *H. pylori* binding to the gastric mucosal surface via the Lewis B blood group antigen (*Leb*), promoting persistent colonization and enhancing CagA translocation, leading to host cellular damage [6].

The *babA2*-positivity prevalence in the current study was 73.4%. In Vietnam, data on the *babA2*-positivity prevalence remain limited. However, our findings are consistent with several Asian studies, which reported a *babA2*-positivity prevalence of over 70%, including studies in China (79.8%) [37] and Korea (79%) [35]. The geographical diversity of *babA2* (*+*) genotypes in *H. pylori* strains has been reported, including 70.2% in Ecuador [38], 53% in Argentina [39] and 44% in Costa Rica [40]. Furthermore, our results demonstrated that the majority of *babA2* (+) *H. pylori* strains (97.6%) possessed the *cagA* (*+*) gene (Table 3), which is consistent with studies from other regions [41,42]. The presence of both *babA2*(+) and *cagA*(+) genes in *H. pylori* strains may confirm the role of BabA in enhancing CagA translocation [6].

The *H. pylori* infection and pathogenesis require the involvement of multiple virulence factors [8], leading to increased attention to the role of virulence gene combinations in *H. pylori* infections. CagA and VacA are considered the two primary toxins of *H. pylori*, while BabA facilitates CagA translocation into host gastric cells, interacting synergistically to enhance persistent colonization and gastric epithelial damage [16]. Interestingly, our findings highlighted a high prevalence of highly virulent combinations, with *cagA*(+)/*babA2*(+)/*vacA*s1m1 and *cagA*(+)/*babA2*(+)/*vacA*s1m2 accounting for 44.4 and 28.4 %, respectively. The triple-virulent combinations of *H. pylori cagA*(+)/*babA2*(+)/*vacA*s1 have been reported in a Cuban study (56.2%) [41]*.* These combinations further explain the capacity of *H. pylori* strains to induce significant damage to the gastric mucosa.

When evaluating each *H. pylori* virulence gene of *cagA*, *vacA* and *babA2* separately in multivariable logistic analysis after adjusting age group and gender, no significant association was observed between *vacA s1m1*, *s1m2* or *babA2* (+) genes and gastroduodenal diseases; only the *cagA* (*+*) gene was associated with an increased odds of peptic ulcer disease. In East Asian countries, where most *H. pylori* strains carry the *cagA* (*+*) gene, the association between *cagA* (*+*) strains and gastroduodenal diseases has been controversial [11,43, 44]. A meta-analysis conducted across several Southeast Asian countries found that the *cagA* gene was associated with an increased risk of peptic ulcer disease, whereas studies in Thailand and China reported no such association between *cagA* (+) strains and gastroduodenal diseases [32,33, 45].

Furthermore, although previous studies have reported different toxicity levels among *vacA* genotypes, where the s1m1 subtype exhibits the highest vacuolating activity, s1m2 demonstrates intermediate activity and s2m2 shows no vacuolating activity [6], our study did not identify any association between *vacA* genotypes and gastroduodenal diseases.

The *babA2* gene encodes the first known adhesin of *H. pylori*, but its association with gastroduodenal diseases has been inconsistent [17,32, 46, 47]. Our findings align with several Asian studies that have reported no significant association between *babA2* (*+*) and gastroduodenal diseases [32,48]. However, this contrasts with a meta-analysis reporting a significant association between the *H. pylori babA2* (+) strains and peptic ulcer disease [17] or a Chinese study finding an association between the *babA2* gene and atrophic gastritis and intestinal metaplasia in the antrum [37]. Overall, the inconsistency between our study and worldwide studies of the association of separate *cagA*, *vacA* sm or *babA2* with gastroduodenal diseases suggests that individual virulence genes may not be sufficient predictors of gastroduodenal disease severity.

Our analysis of the combination of three genes revealed that the *cagA*(+)/ *babA2*(+)/*vacA*s1m2 genotype was associated with an increased odds of peptic ulcer disease in male patients and chronic gastritis with precancerous lesions in female patients, representing a key finding of our study. Since multiple *H. pylori* virulence factors contribute to pathogenesis, their synergistic effects on the development of *H. pylori*-induced gastroduodenal diseases have been increasingly considered [6,6]. Our findings provide initial evidence supporting this hypothesis. CagA and VacA are the two main toxins of *H. pylori*, and their combined effects on gastroduodenal disease severity have been well established [12]. Although *vacA* s1m1 has been recognized as having the highest vacuolating ability, our study identified *vacA* s1m2 as playing a more significant role in the synergistic effect with *cagA* (+) and *babA2* (+) strains. A study on the functional properties of *H. pylori* VacA variants suggested that the *vacA* m2 allele exhibits cell type-specific activity and may contribute to gastric cancer or peptic ulcer disease, particularly when coexisting with the *cag* pathogenicity island (*cag*PAI) [49]. Additionally, a meta-analysis revealed a relationship between *H. pylori vacA* m-region genotypes and *cagA* status, indicating an increased risk of developing peptic ulcer disease in Southeast Asian populations [45]. Furthermore, the *babA2* gene encodes the BabA protein, a crucial adhesin that facilitates persistent bacterial colonization and enhances CagA translocation into gastric cells. This interaction explains the synergistic effect of *cagA* and *babA2* in the development of gastroduodenal diseases [6]. Limited research has documented the association between the *cagA*(+)/*vacA*s1m2/*babA2*(+) combination and gastroduodenal diseases, while an Indian investigation indicated that the *cagA*(+)/*vacA*s1m1/*babA2*(+) combination was not associated with disease status [50]. In Asian countries, where most *H. pylori* strains carry the *cagA* gene and *vacA* s1 regardless of gastroduodenal disease severity, our findings on a gene combination associated with peptic ulcer disease and gastric precancerous lesions may serve as a valuable basis for future large-scale research. Identifying a highly predictive bioindicator of highly virulent *H. pylori* strains significantly associated with severe gastroduodenal diseases could aid in selecting patients for more aggressive treatment strategies.

## Conclusions

In summary, our data identified the great prevalence of gene combinations of *cagA*(+)*/babA2*(+)*/vacAs1m1 (*44.4%) and *cagA*(+)*/babA2*(+)*/vacAs1m2* (28.4%) among Vietnamese *H. pylori* strains. The gene combination of *cagA*(+)/*babA2*(+)/*vacAs1m2* was associated with an increased odds of peptic ulcer disease in male patients and gastric precancerous lesions in female patients.

## Abbreviations

aOR: adjusted odds ratio
*babA2*: blood group antigen-binding adhesion 2
*cagA* (+): *cagA*-positive gene
*cagA*: cytotoxin-associated gene A
*cagA* (−): *cagA*-negative gene
*cag*PAI empty (+): *cag*PAI empty site-positive
cagPAI empty (−): cagPAI empty site-negative
CI: confidence interval
m: middle (m) region
s: signal region
SHP2: SH2 containing protein tyrosine phosphatase-2
*vacA*: vacuolating cytotoxin A
*vacA* m1: *vacA* middle region 1 allele
*vacA* m2: *vacA* middle region 2 allele
*vacA* s1: *vacA* signal region 1 allele
*vacA* s2: *vacA* signal region 2 allele
*vacA* s1m1: vacA with signal region 1 allele (s1) and middle region 1 allele (m1)
*vacA* s1m2: *vacA* with signal region 1 allele (s1) and middle region 2 allele (m2)
*vacA* s2m2: *vacA* with signal region 2 allele (s2) and middle region 2 allele (m2)
